# Acquired factor XIII deficiency in adult patients during ECMO: a prospective observational study

**DOI:** 10.1038/s41598-025-26452-9

**Published:** 2025-11-07

**Authors:** Matthias Noitz, Dennis Jenner, Thomas Tschoellitsch, Roxane Brooks, Romana Erblich, Niklas Krenner, Tina Tomić-Mahečić, Martin W. Dünser, Andreas Zierer, Jens Meier

**Affiliations:** 1 Department of Anesthesiology and Intensive Care Medicine, Kepler University Hospital GmbH, Johannes Kepler University, Krankenhausstraße 9, 4020 Linz and Altenberger Strasse 69, 4040 Linz, Austria; 2 Medical Faculty, Johannes Kepler University Linz, Altenberger Strasse 69, 4040 Linz, Austria; 3 Department of Cardiothoracic and Vascular Surgery, Kepler University Hospital GmbH, Johannes Kepler University Linz, Krankenhausstraße 9, 4020 Linz and Altenberger Strasse 69, 4040 Linz, Austria; 4 Department of Anesthesiology and Intensive Care Medicine, University Hospital Center Zagreb - Rebro, Zagreb, Croatia

**Keywords:** Transfusion, Haemoglobin, Bleeding, Major bleeding event, FXIII deficiency, ECMO, Diseases, Health care, Medical research, Risk factors

## Abstract

Data on factor XIII (FXIII) activity in patients on extracorporeal membrane oxygenation (ECMO) are scarce. In this prospective, observational, single-centre study, we determined the rate of acquired FXIII deficiency in 44 adults during ECMO. We evaluated the relationship between minimum FXIII activity, haemoglobin levels, transfusion requirements, as well as the occurrence of major bleeding events. Descriptive statistical methods, bivariate correlation and receiver operating characteristic curve analyses were applied. Acquired FXIII deficiency (FXIII activity < 70%) was found in 41/44 patients [93.2% (95% CI 85.7–100%)] during ECMO. The minimum FXIII activity was correlated with minimum haemoglobin levels (Spearman-*rho*, 0.331, 95% CI 0.02–0.57, *p* = 0.03), packed red blood cell transfusion requirements (Spearman-*rho*, − 0.32, 95% CI − 0.58 to 0.00, *p* = 0.03), but not the occurrence of major bleeding events (*p* = 0.32, Hodges-Lehmann difference − 4.0%, 95% CI − 15.0 to 5.0%) during ECMO. The area under the receiver operating characteristic curve for minimum FXIII activity to predict the occurrence of major bleeding events during ECMO was 0.602 (95% CI 0.375–0.828). The lowest FXIII activity to predict a major bleeding event was 51.0% and 55.5% (Youden index for both, 0.27), respectively. In conclusion, acquired FXIII deficiency is highly frequent in adult ECMO patients. Lower FXIII activity showed modest correlations with nadir haemoglobin and pRBC exposure, but did not discriminate patients with major bleeding. Larger studies are needed to determine whether routine FXIII monitoring and targeted replacement improve outcomes during ECMO.

**Supplementary Information:**

The online version contains supplementary material available at 10.1038/s41598-025-26452-9.

## Introduction

Extracorporeal membrane oxygenation (ECMO) is a life-support strategy for patients with refractory cardiac and/or respiratory failure. Over the last decades, it has been widely adopted both in veno-venous (VV) and veno-arterial (VA) configuration^1,2^. Despite advances in technology, circuit design, and anticoagulation strategies, ECMO remains associated with a high incidence of complications substantially contributing to morbidity and mortality^3,4^. Clinically significant bleeding occurs in 30–60% of patients on ECMO, while up to 50% of ECMO patients develop thromboembolic complications^3,4^.

Haemostatic derangements observed during ECMO, collectively referred to as ECMO-induced or ECMO-associated coagulopathy, are complex and multifactorial in their pathogenesis. They arise from the interplay between critical illness-induced haemostatic dysfunction, systemic inflammation, continuous exposure of blood to artificial surfaces, and the use of systemic anticoagulation to prevent circuit thrombosis^5,6^. The resulting coagulopathy affects both primary (*e.g.*, thrombocytopenia, qualitative platelet dysfunction, acquired von Willebrand syndrome) and secondary haemostasis (*e.g.*, consumption of coagulation factors, contact pathway activation), dysregulates fibrinolysis, and induces immunothrombosis^5–9^.

Coagulation factor XIII (FXIII) is a tetrameric plasma transglutaminase, that catalyses the covalent cross-linking of fibrin monomers during the final phase of the coagulation cascade, thereby stabilizing the fibrin network, increasing mechanical clot resistance and preventing premature degradation^10,11^. Acquired FXIII deficiency has been associated with an increased perioperative bleeding risk, higher transfusion requirements, and increased surgical re-exploration rates across various clinical populations and scenarios, including medical, trauma and cardiac surgery patients^12–14^. Within the context of ECMO, the role of FXIII has not yet been adequately characterized. Case reports and smaller, mainly retrospective studies suggest that acquired FXIII deficiency also occurs in adult and paediatric ECMO patients^15–17^. A single-centre, retrospective analysis recently identified low FXIII activity levels during ECMO as an independent risk factor for major bleeding events and blood transfusion^18^.

In this study, we aimed to determine the rate of acquired FXIII deficiency in adult ECMO patients and investigate the relationship between minimum FXIII activity and haemoglobin levels, transfusion requirements, and the occurrence of major bleeding events.

## Methods

This analysis was designed as a prospective, observational, single-centre cohort study. Between December 1, 2022, and October 31, 2024, it was conducted in a 22-bed, level III intensive care unit (ICU) of the Department of Anaesthesiology and Critical Care Medicine at Kepler University Hospital, a tertiary academic centre and ELSO (Extracorporeal Life Support Organization)—certified ECMO referral facility located in Linz/Austria. The study protocol was approved by the Ethics Committee of the Medical Faculty of the Johannes Kepler University Linz on July 12, 2022 (reference number: 1008/2022). Written informed consent was obtained from all study patients or their next-of-kin. The research described in this study was conducted in accordance with relevant guidelines and regulations, as well as the principles outlined in the Declaration of Helsinki. This manuscript was drafted in accordance with the STROBE (Strengthening the Reporting of Observational Studies in Epidemiology) checklist for cohort studies^19^.

### Study population

All patients requiring VV- or VA-ECMO were eligible for study enrolment. Exclusion criteria comprised age < 18 years, pre-existing hereditary haemorrhagic or thrombophilic disorders, ECMO initiated as part of extracorporeal cardiopulmonary resuscitation using the CARL® system (Controlled Automated Reperfusion of the Whole Body, RESUSCITEC GmbH, Freiburg, Germany), ECMO duration < 24 h, and cases in which ECMO was applied as temporary right ventricular support following implantation of a left ventricular assist device.

### ECMO circuit, anticoagulation and haemostatic management during ECMO therapy

ECMO therapy was provided using the Xenios Console® system (Xenios AG, Fresenius Medical Care, Heilbronn, Germany), a multifunctional platform capable of cardiac and pulmonary support. Heparin-albumin-coated tubing systems were used (Novalung XLung kit 230®, Xenios AG, Fresenius Medical Care, Heilbronn, Germany), and vascular access was achieved with heparin-albumin-coated cannulas (Getinge AB®, Gothenburg, Sweden). Continuous infusion of unfractionated heparin was the first-line anticoagulation therapy, with a target activated partial thromboplastin time (aPTT) range of 50–60 s. If this target range could not be achieved with daily heparin doses exceeding 40,000 IU, or in case of suspected heparin—induced thrombocytopenia, anticoagulation was switched to continuous infusion of argatroban. In addition, epoprostenol was continuously infused into the oxygenator at an hourly rate of 0.005–0.01 mg to mitigate platelet activation. Packed red blood cells (pRBCs) were transfused to maintain a target haemoglobin concentration of 7–9 g/dL. Platelet transfusions were administered aiming for platelet counts ≥ 50 G/L in nonbleeding patients and ≥ 100 G/L in bleeding patients^20^. Plasma transfusions, coagulation factor concentrates (including FXIII concentrates), or antifibrinolytic agents were administered on an individualized basis guided by the clinical context and the judgment of the multidisciplinary critical care team. Per protocol, administration of FXIII concentrates was restricted to patients with FXIII activity < 70% and either a high risk of bleeding (e.g. surgical intervention), active bleeding or following the occurrence of a bleeding event.

### Study variables and data collection

The following study-related variables were collected for all patients: age, sex, body mass index, comorbidities, prior surgery, indication and configuration of ECMO, anticoagulation therapy, the Simplified Acute Physiology Score II (SAPS II) at ICU admission, most aberrant levels of lactate, creatinine, and bilirubin, as well as lowest platelet count during ECMO, complications (haemorrhagic and thromboembolic events), number of pRBC units transfused during ECMO, ECMO duration, need for bridging to an assist device or transplantation, ICU length of stay, as well as ICU, 28-day, and hospital mortality.

FXIII activity was measured using the Berichrom® FXIII chromogenic assay (Siemens Healthineers, Marburg, Germany) performed on a Sysmex CN-6000 analyzer. Blood was collected in 3.2% sodium-citrate tubes (BD Vacutainer®) and blood samples were processed in the central institutional laboratory of Kepler University Hospital and analyzed within 60 to 90 min. The laboratory reference range for FXIII activity was 70–140%. FXIII activity was measured at ECMO initiation and at 48-h intervals throughout the course of ECMO and recorded until day 14.

Viscoelastic testing was performed during ECMO every 48 h using the point-of-care ROTEM® sigma (Werfen, Barcelona, Spain). Bedside measurements were obtained within one hour of blood sampling. The following four ROTEM assays were investigated: EXTEM, INTEM, FIBTEM, and APTEM. Within each assay, clotting time (CT), clot formation time (CFT), amplitudes at 5, 10, 20 and 30 min (A5/A10/A20/A30), maximum clot firmness (MCF), and lysis indices at 30 and 60 min (LI30/LI60) were recorded.

Standard coagulation parameters including activated partial thromboplastin time (aPTT), prothrombin time, antithrombin III, and unfractionated heparin (UFH) anti-Xa activity were recorded every 8 h. Platelet count and D-dimer levels were measured daily. Von Willebrand diagnostics were performed on day 3 after ECMO initiation.

### Definitions

Acquired FXIII deficiency was defined as a plasma FXIII activity level < 70%, based on the minimum activity measured during ECMO. This threshold reflects established reference ranges for normal FXIII activity (typically 70–140%), is consistent with the definitions applied in previous studies^12,18,21^ and differs from hereditary FXIII deficiency^22^. We further classified FXIII deficiency as mild (FXIII activity, 50–69%), moderate (FXIII activity, 30–49%) or severe (FXIII activity, < 30%). Major bleeding events were defined in line with the Extracorporeal Life Support Organization criteria^23^, including any of the following:

- A decrease in haemoglobin levels ≥ 2 g/dL within 24 h.
- Blood loss exceeding 20 mL/kg over 24 h.
- Transfusion requirement of one or more 10 ml/kg pRBC transfusions within 24 h.
- Evidence of retroperitoneal, pulmonary, or central nervous system bleeding; or
- Any bleeding event requiring surgical intervention.

Thromboembolic events were defined as the clinical or radiographic diagnosis of pulmonary embolism, deep vein thrombosis, arterial thrombosis, or ischemic stroke. In addition, suspected or visually confirmed thrombosis within the ECMO circuit (e.g., oxygenator or pump head) or within venous or arterial ECMO cannulas was classified as a thromboembolic event.

### Study goals

The primary goal of this study was to determine the rate of FXIII deficiency in patients on ECMO. The secondary study goal was to evaluate the relationship between minimum FXIII activity and minimum haemoglobin levels, pRBC transfusion requirements, as well as the occurrence of major bleeding events during ECMO. Furthermore, we sought to identify a cut-off level for the lowest FXIII activity, which predicts the occurrence of major bleeding events during ECMO.

### Statistical analysis

Assuming an incidence of FXIII deficiency during ECMO of 69%, as identified in a previous retrospective cohort study^18^, we calculated that (compared to a null hypothesis of 20%) inclusion of at least 40 patients would yield a high power (> 99%) at an alpha-level of 0.05 in order to determine the true incidence of FXIII deficiency in this patient population. Expecting a 10% drop-out rate, we enrolled 44 patients.

Following plausibility control of the study database, all statistical analyses were performed using the R® statistical software package (Version 4.4.3; R Core Development Team, Vienna, Austria). No imputation methods were used to compensate for missing values. Shapiro Wilk tests were used to evaluate normality distribution of continuous study variables. Demographic characteristics and clinical parameters were reported using descriptive statistical methods. The primary study endpoint was given as the rate of study patients, who developed a FXIII deficiency during ECMO. Ninety-five percent confidence intervals (95% CI) were calculated to report the precision of the primary endpoint. We also conducted sensitivity analyses to evaluate the robustness of our findings by testing the primary study endpoint in patient groups based on their ECMO configuration (VV vs. VA) and surgical status (surgery vs. no surgery prior to ECMO) as well as FXIII supplementation status to address time-varying FXIII exposure and to mitigate confounding-by-indication. The relationship between minimum FXIII activity and minimum haemoglobin levels, as well as pRBC transfusion requirements, was determined using bivariate correlation analyses with Spearman-rho correlation coefficients. The relationship between minimum FXIII activity and the occurrence of major bleeding events was evaluated by comparing minimum FXIII activity between study patients with and without a major bleeding event using Mann Whitney U-tests. A receiver operating characteristic curve analysis was applied to determine the value of minimum FXIII activity to predict the occurrence of a major bleeding event during ECMO. Optimal thresholds were identified based on the Youden index. A two-sided *p*-value < 0.05 was considered to indicate statistical significance. Categorical variables are presented as absolute numbers with percentages, while continuous variables are reported as median values with interquartile ranges.

## Results

**Fig. 1:**
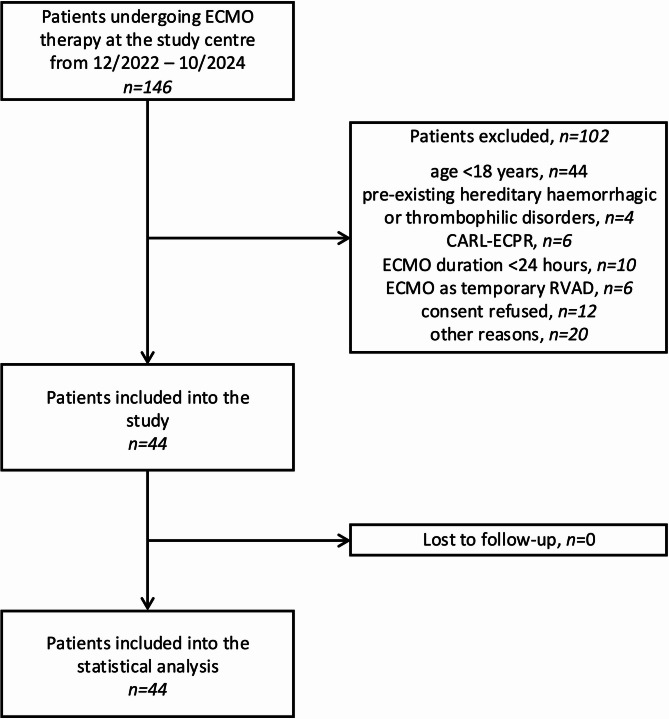
Study flow diagram. CARL, controlled automated reperfusion of the whole body; ECMO, extracorporeal membrane oxygenation; ECPR, extracorporeal cardiopulmonary resuscitation; RVAD, right ventricular assist device.

**Fig. 2:**
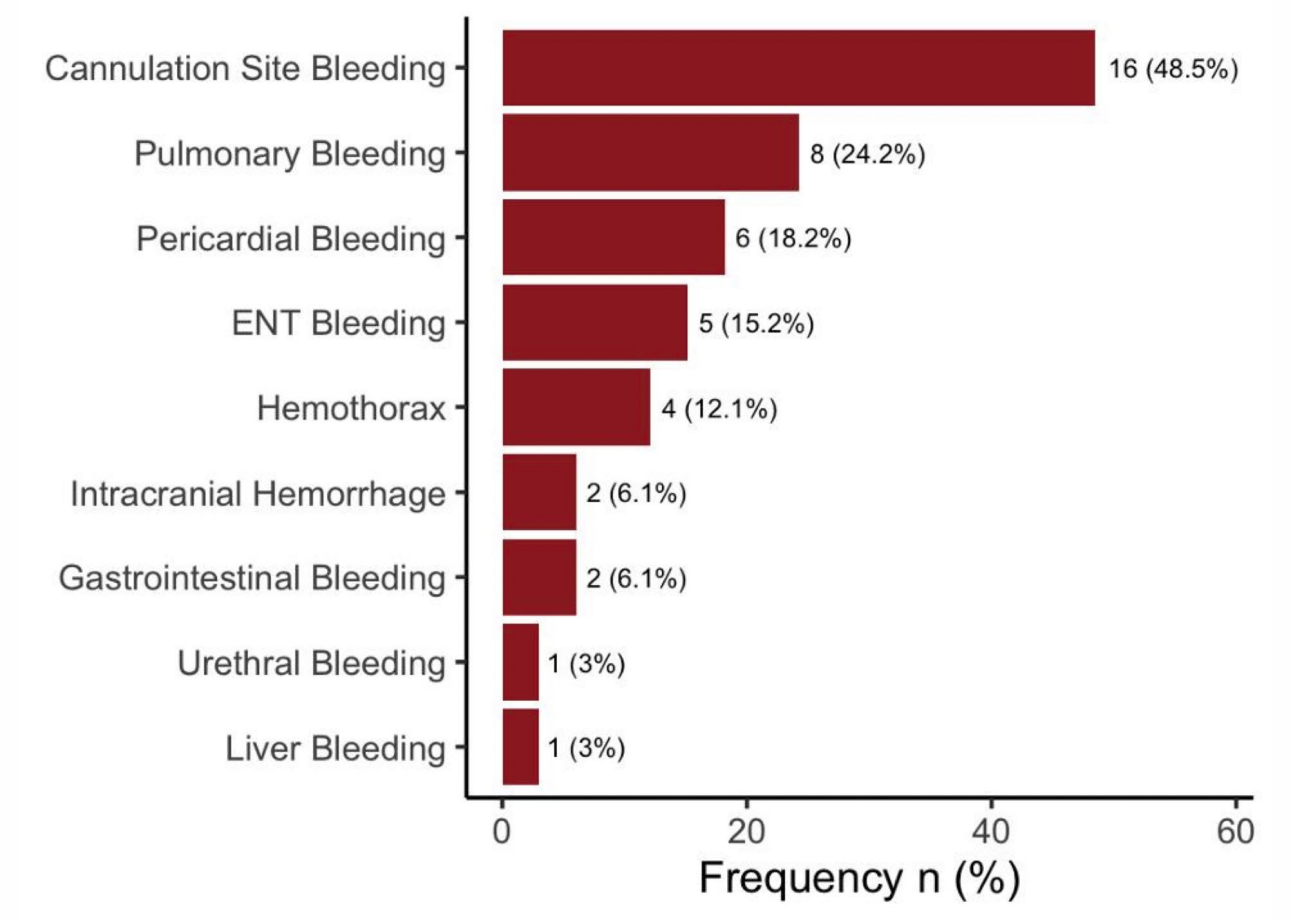
Frequency and type of bleeding complications. Bars show n (%) for each bleeding site. Percentages are relative to patients with any major bleeding (n = 33). Patients could meet > 1 site category, therefore totals exceed 100%. ENT, ear, nose and throat bleeding.

146 patients undergoing ECMO therapy at the study center during the observation period were screened. 102 were excluded (age < 18 years, n = 44; hereditary haemorrhagic/thrombophilic disorders, n = 4; CARL-ECPR, n = 6; ECMO duration < 24 h, n = 10; temporary RVAD after LVAD implantation, n = 6; consent refused, n = 12; other reasons, n = 20), leaving 44 subjects (30.1%) to be enrolled into the final statistical analysis (Fig. 1; Supplementary Table S1). No patient was lost to follow-up. Table 1 summarizes demographic and clinical characteristics of the study population. While 32 (72.7%) study patients received VA-ECMO, 12 (27.3%) subjects underwent ECMO in a VV-configuration. 2 patients, who had initially been supported in VV-configuration, were subsequently converted to VA-mode because of right heart failure. Overall, 21 (47.7%) study patients underwent surgery prior to ECMO initiation. 33 (75%) patients experienced one or more major bleeding events during ECMO (Fig. 2). Among patients with a major bleeding event, the most frequent bleeding sites were cannulation site in 16 (48.5%) patients, pulmonary bleeding in 8 (24.2%) patients and pericardial bleeding in 6 (18.2%) patients respectively. The transfusion criterion (≥ 10 mL/kg pRBC within 24 h) was the most frequently fulfilled ELSO sub-criterion in 24 (72.7%), followed by a haemoglobin drop ≥ 2 g/dL/24 h in 17 (51.5%) and need for surgical intervention due to bleeding in 8 (24.2%) study patients respectively. A detailed description of major-bleeding sub-criteria and timing to first major bleeding event is provided in Supplementary Tables S2 and S3. Further patient outcomes are presented in Table 2.

**Table 1:** Demographic and clinical characteristics of the patient population.

*N*		44
Age	years	59 (51.5–67)
Male sex	n (%)	32 (72.7)
Body mass index	kg/m^2^	26.6 (24.1–30)
Comorbid conditions		
Dyslipidaemia	n (%)	16 (36.4)
Coronary artery disease	n (%)	15 (34.1)
Arterial hypertension	n (%)	14 (31.8)
Cardiomyopathy/CHF	n (%)	13 (29.5)
Diabetes mellitus	n (%)	10 (22.7)
Chronic respiratory disease	n (%)	8 (18.2)
Immunosuppression	n (%)	7 (15.9)
Chronic kidney disease	n (%)	6 (13.6)
Chronic liver disease	n (%)	4 (9.1)
Surgery before ECMO therapy	n (%)	21 (47.7)
cardiothoracic surgery	n (%)	19 (90.5)
abdominal surgery	n (%)	2 (9.5)
Indication for ECMO		
Postcardiotomy shock	n (%)	19 (43.2)
Viral pneumonia	n (%)	7 (15.9)
Cardiogenic shock	n (%)	6 (13.6)
ECPR	n (%)	5 (11.4)
Secondary ARDS	n (%)	4 (9.1)
Bacterial pneumonia	n (%)	2 (4.5)
Hypercapnic respiratory failure	n (%)	1 (2.3)
ECMO configuration^1^		
VV ECMO	n (%)	12 (26.1)^1^
VA ECMO	n (%)	34 (73.9) ^1^
-cross-over from VV to VA ECMO	n (%)	2(4.3)^1^
Anticoagulation during ECMO		
unfractionated heparin	n (%)	44 (100)
additional epoprostenol	n (%)	44 (100)
cross-over to argatroban	n (%)	3 (6.8)
SAPS II Score	Points	52 (39–59)
Organ dysfunction during ECMO		
Maximum lactate levels	mmol/L	4.2 (2.7–10.3)
Maximum creatinine concentration	µmol/L	159.1 (106.1–265.2)
Maximum bilirubin concentration	µmol/L	176.8 (123.8–335.9)
Minimum platelet count	G/L	51 (37–72)

*ARDS* acute respiratory distress syndrome;* CHF* congestive heart failure;* ECMO* extracorporeal membrane oxygenation;* ECPR* extracorporeal cardiopulmonary resuscitation;* SAPS* simplified acute physiology score;* VA* veno-arterial;* VV* veno-venous.

Data are given as median values with interquartile ranges, if not otherwise indicated. ^1^Data refer to 46 ECMO runs in 44 individual patients.

**Table 2:** Patient outcomes.

*N*		44
Complications during ECMO		
Major bleeding event	n (%)	33 (75.0)
Thromboembolic event	n (%)	12 (27.3)
Number of pRBC units transfused during ECMO	Units	10 (6–19)
Early pRBC transfusion exposure (< 24 h after ECMO start)	Units	2 (0–3)
Later pRBC transfusion exposure (> 24 h hours after ECMO start	Units	8 (6–16)
Duration of ECMO	Days	9 (6–13)
Bridging to assist device	n (%)	3 (6.8)
Bridging to transplant	n (%)	4 (9.1)
Intensive care unit length of stay	Days	16 (13–25)
Mortality		
Intensive care unit mortality	n (%)	10 (22.7)
28-day mortality	n (%)	10 (22.7)
Hospital mortality	n (%)	11 (25.0)

ECMO, extracorporeal membrane oxygenation; pRBC, packed red blood cells.

Data are given as median values with interquartile ranges, if not otherwise indicated.

Descriptive coagulation parameters and ROTEM values are provided in Tables 3 and 4. Correlation analyses between minimum FXIII activity and EXTEM CT showed only weak and borderline inverse associations [Spearman ρ =  − 0.31, (95% CI − 0.58–0.03), *p* = 0.049, *n* = 41], but no significant correlations with other viscoelastic metrics.

**Table 3:** Coagulation parameters during ECMO therapy.

Coagulation parameter	Unit	Minimum	Maximum
Platelet count	G/L	51 (38–70)	145 (117–195)
Antithrombin III activity	%	54 (44–69)	113 (99–128)
D-dimer	µg/mL	1.7 (0.9–4.1)	21 (7.6–35.2)
Fibrinogen	mg/dL	222 (182–369)	704 (594–900)
aPTT	Seconds	30.8 (25.8–33.0)	59.6 (49.2–75.3)
Prothrombin time	Seconds	53.5 (41.0–68.5)	119 (101–138)
UFH anti-Xa activity	IU/mL	0.1 (0.1–0.1)	0.3 (0.2–0.45)
FVIII activity	%	253 (162–34)	394 (267–450)
vWF antigen	%	252 (196–372)	303 (208–392)
vWF activity	%	183 (132–244)	209 (161–276)
FVIII/vWF Ag ratio	–	0.9 (0.7–1.2)	0.9 (0.7–1.3)
Von Willebrand factor ratio	–	0.8 (0.7–0.8)	0.8 (0.7–0.8)

*aPTT* activated partial thromboplastin time;* FVIII* coagulation factor VIII;* UFH* unfractionated heparin;* vWF* von Willebrand factor;* vWF Ag* von Willebrand factor antigen. Values represent the lowest and highest recorded measurements per patient during ECMO support. Data are presented as median values + IQR.

**Table 4:** ROTEM parameters during ECMO therapy.

ROTEM parameters	Unit	Median (IQR)
APTEM CT	Seconds	72.5 (64.8–84.3)
APTEM CFT	Seconds	70.5 (47.3–92)
APTEM A5	mm	45.5 (40–57.5)
APTEM A10	mm	63 (57.1–67.8)
APTEM A20	mm	63.0 (57.1–67.8)
APTEM A30	mm	63.5 (58–68.5)
APTEM MCF	mm	64.5 (59–69.5)
APTEM LI30	(%)	100 (100–100)
APTEM LI60	(%)	99 (97–100)
EXTEM CT	Seconds	75 (68–83)
EXTEM CFT	Seconds	67.5 (59–91)
EXTEM A5	mm	48 (41–52)
EXTEM A10	mm	62.5 (58–68)
EXTEM A20	mm	62.5(58.0–68.0)
EXTEM A30	mm	63 (59.5–69)
EXTEM MCF	mm	63 (59.5–69)
EXTEM LI30	(%)	100 (100–100)
EXTEM LI60	(%)	98 (96.5–99)
FIBTEM CT	Seconds	75 (68–84)
FIBTEM CFT	Seconds	142(64–518.3)
FIBTEM A5	mm	17(12–25)
FIBTEM A10	mm	20(14.5–28)
FIBTEM A20	mm	20 (14.5–28.0)
FIBTEM A30	mm	21 (15.5–29)
FIBTEM MCF	mm	21 (15.5–29)
FIBTEM LI30	(%)	100 (100–100)
FIBTEM LI60	(%)	100 (100–100)
INTEM CT	Seconds	227(197–266)
INTEM CFT	Seconds	91 (56–104)
INTEM A5	mm	40.5 (37–49)
INTEM A10	mm	51(47–59)
INTEM A20	mm	57(54.0–65.5)
INTEM A30	mm	59 (56–67.5)
INTEM MCF	mm	59 (56–67.5)
INTEM LI30	(%)	100 (100–100)
INTEM LI60	(%)	98.5 (97–99.8)

*A5* amplitude at 5 min;* A10* amplitude at 10 min;* A20* amplitude at 20 min;* A30* amplitude at 30 min;* CT* clotting time;* CFT* clot formation time;* MCF* maximum clot firmness;* LI30* lysis index after 30 min;* LI60* lysis index after 60 min.

### Primary study endpoint: rate of FXIII deficiency

**Fig. 3:**
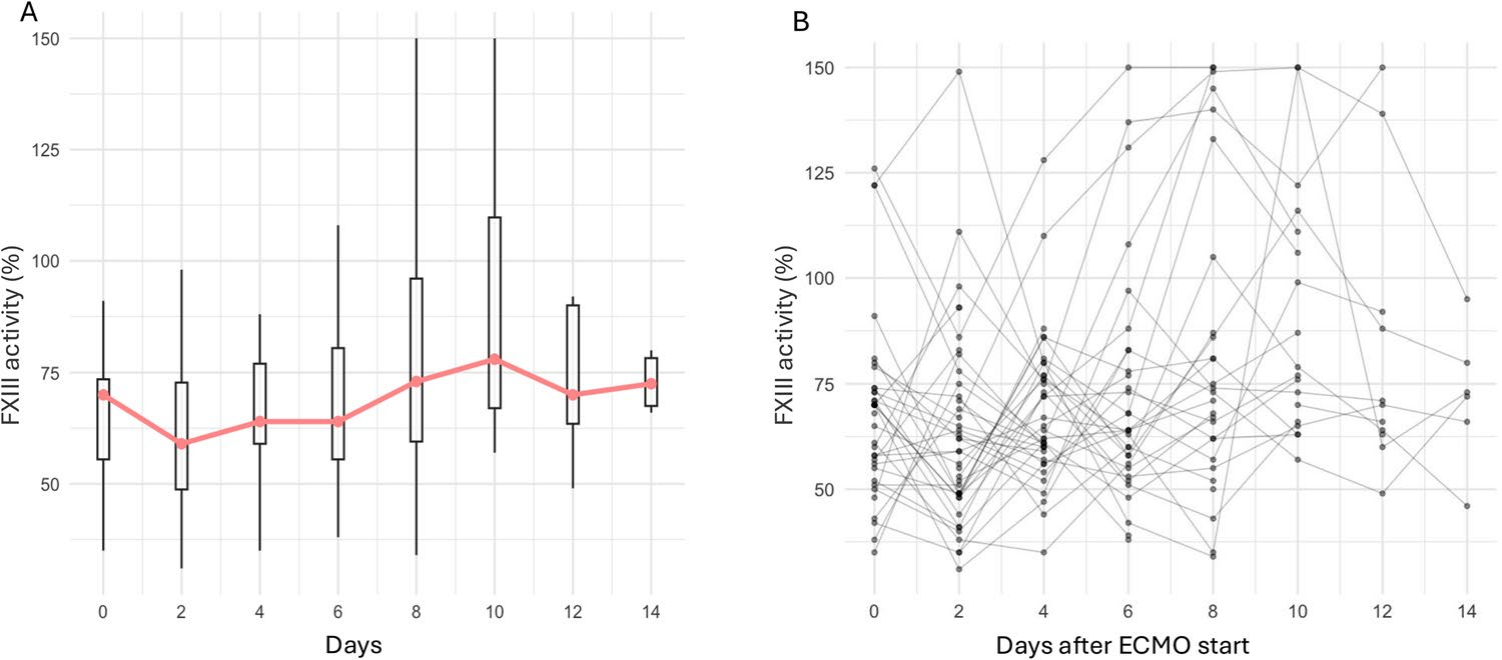
FXIII activity over time during ECMO. (**A**) Distribution of FXIII activity (%) at scheduled assay days after ECMO start. (**B**) Individual trajectories for all patients.

In 41 out of 44 study patients, a plasma FXIII activity < 70% was observed during ECMO. This corresponded to a rate of acquired FXIII deficiency of 93.2% (95% CI 85.7–100%). Acquired FXIII deficiency was classified as mild in 18 patients (43.9%), moderate in 23 patients (56.1%), and severe in no patient (0%). Acquired FXIII deficiency was present in 17 study patients [38.6% (95% CI 24.4–54.5%)] at ECMO initiation [mild, *n* = 5; moderate, *n* = 12; severe, *n* = 0]. FXIII activity over time during ECMO therapy is illustrated in Fig. 3. The rate of acquired FXIII deficiency was 91.7% (95% CI 61.5–99.8%) (11/12) in study patients on VV-ECMO mode and 93.8% (95% CI 79.2–99.2%) (30/32) in those on VA-ECMO (*p* = 0.81; Chi^2^-test). Study patients, who underwent surgery before initiation of ECMO, exhibited acquired FXIII deficiency in 95.2% (95% CI 76.2–99.9) of cases (20/21), while patients without surgery before ECMO had FXIII deficiency in 91.3% (95% CI 72.0–98.9%) (21/23) (*p* = 1.00; Chi^2^-test). 35 of 41 patients (85.4%) with an acquired FXIII deficiency received FXIII concentrates at a median dose of 2500 (IQR 1250–2500) IU during ECMO. First FXIII concentrate substitution occurred at 2.0 (IQR 2.0–4.0) days after ECMO start, while the first major bleeding event occurred at 1.0 (IQR 0.5–3.0) days. Supplementary Figure S1 displays per-patient timing of FXIII assays, first FXIII substitution, and first major bleeding event.

### Secondary study endpoints

**Fig. 4:**
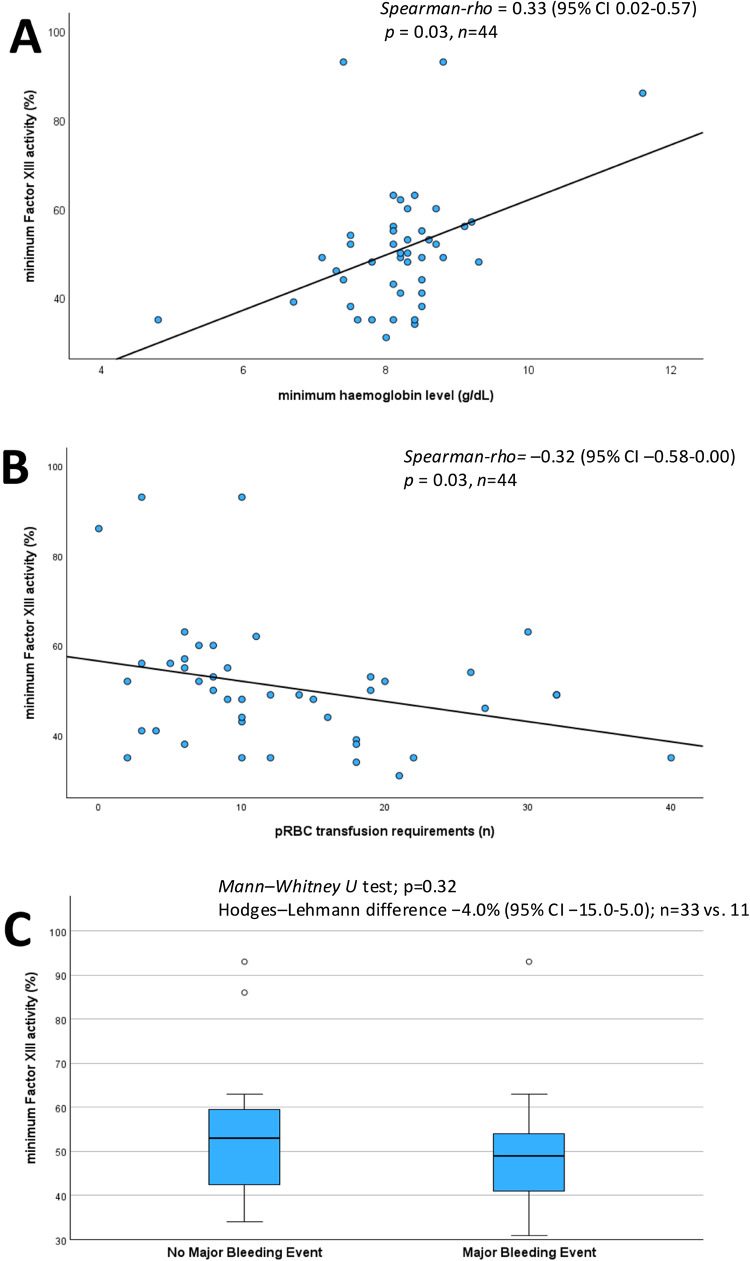
Relationship between minimum factor XIII activity and (**A**) minimum haemoglobin levels, (**B**) number of packed red blood cells transfused, and (**C**) the occurrence of major bleeding events during ECMO therapy. (**A**/**B**) Correlations assessed with Spearman’s ρ. 95% confidence interval (CI), *p*-values and sample size (*n*) are reported. (**C**) Group comparison by Mann–Whitney U. Effect size reported as Hodges–Lehmann median difference with 95% CI.* pRBC* packed red blood cells.

**Fig. 5:**
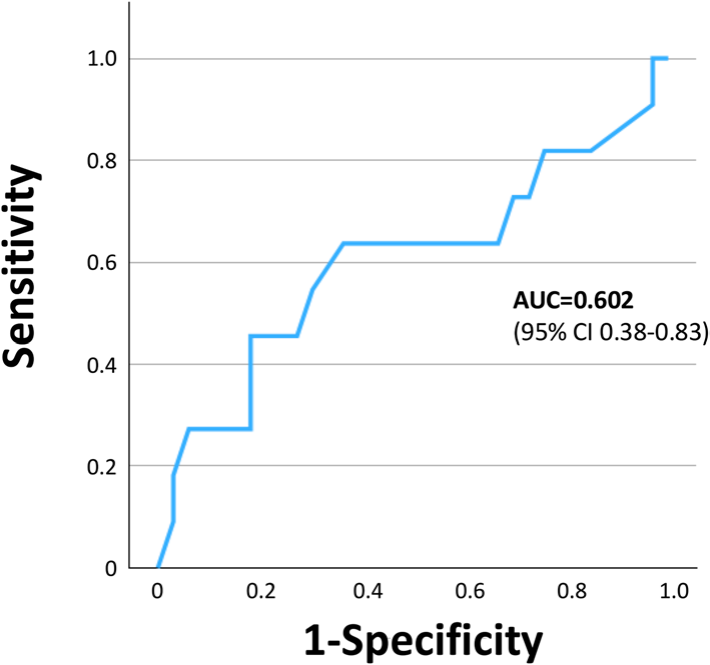
Receiver operating characteristic curve on the discriminative value of minimum FXIII activity to predict the occurrence of a major bleeding event. AUC, area under the curve; CI, confidence interval.

Minimum FXIII activity showed a positive association with nadir haemoglobin [Spearman ρ = 0.33, (95% CI 0.02–0.57), *p* = 0.028*, n* = 44] and was inversely associated with total pRBC units transfused [Spearman ρ = –0.32, (95% CI –0.58 to 0.00), *p* = 0.034, *n* = 44] (Fig. 4A and B). However, minimum FXIII activity did not differ between patients with versus without a major bleeding event [median 49.0% (IQR 41.0–54.0) vs. 53.0% (42.5–59.5), Hodges–Lehmann difference − 4.0% (95% CI − 15.0 to 5.0%), *p* = 0.32, n = 33 vs. 11] (Fig. 4C).The area under the receiver operating characteristic curve for the minimum FXIII activity to predict the occurrence of a major bleeding event during ECMO was 0.602 (95% CI 0.375–0.828) (Fig. 5). The lowest FXIII activity to predict a major bleeding event was 55.5%, yielding a sensitivity of 81.8% and a specificity of 45.5%. A lower threshold of 51.0% provided a more balanced sensitivity and specificity of 63.6% each, with both exploratory cut-offs demonstrating an identical limited specificity (Youden Index of 0.27).

In a sensitivity analysis restricted to FXIII activity levels measured before occurrence of FXIII concentrate supplementation, pre-supplementation minimum FXIII levels did not differ between patients with (median factor XIII activity 52% [IQR 43–56%]) and without major bleeding events (median factor XIII activity 50% [47–58.5%], *p* = 0.956). Factor XIII activity showed no relevant discrimination for the occurrence of major bleeding during ECMO therapy (AUC 0.51, 95% CI 0.30–0.72). Excluding patients who received FXIII concentrates before their first bleed event yielded similar results (AUC 0.52, 95% CI 0.32–0.73).

### Subgroup analysis: postcardiotomy cohort

In the postcardiotomy subgroup (n = 19), ECMO was started intraoperatively in 13 (68.4%) patients, on the same postoperative day in 3 (15.8%) patients, and on postoperative day 1, 3 and 8 in 1 (5.3%) study patient each. Antifibrinolytics were used in 17 (89.5%) cardiac surgery patients (tranexamic acid 42.1%, aprotinin 47.4%). Intraoperative transfusion and coagulation management included administration of pRBCs in 10 (52.6%) patients [median 4 units (IQR 2–4)], platelets in 7 (36.8%) patients [median 2 units (IQR 1–2)], fresh frozen plasma in 7 (36.8%) patients [median 3 units (IQR 2–6)], prothrombin complex concentrate in 7 (36.8%) patients [median 2,000 international units (IQR 1800–3000)], and fibrinogen concentrate in 9 (47.4%) patients [median 4,000 mg (IQR 3000–5000)] respectively. No patient received intraoperative FXIII concentrate. Median cardiopulmonary bypass time was 206 min (IQR 100–248) and median surgical time 328 min (IQR 248–447). Minimum FXIII activity was not associated with cardiopulmonary bypass duration [Spearman ρ =  − 0.09, (95% CI − 0.64–0.47), *p* = 0.738, *n* = 17].

## Discussion

In this prospective, observational, single-centre cohort study, we found a very high rate of acquired FXIII deficiency of 93.2% among adult patients undergoing ECMO therapy. The 95% confidence interval of 85.7 to 100% suggests that it is highly likely that the true estimate of the primary study endpoint exceeds 85%. A similar rate of acquired FXIII deficiency was detected in study patients on VV- or VA-ECMO, as well as in those with or without surgery before initiation of ECMO. Minimum FXIII activity in our cohort was correlated with minimum haemoglobin levels and the number of pRBC units transfused during ECMO, but not with the occurrence of major bleeding events.

As part of the study protocol, patients with known hereditary haemorrhagic disorders were excluded to ensure a specific focus on acquired alterations. Accordingly, it can be assumed that FXIII deficiency was acquired in our cohort. The observed rate of FXIII deficiency in the present study exceeded that reported in an earlier retrospective, single-centre data collection at our centre^18^. The systematic and prospective screening for acquired FXIII deficiency in the present study is likely the reason for this discrepancy. Applying a systematic protocol to screen for acquired coagulation disorders, Kalbhenn et al*.* reported a similarly high rate of FXIII deficiency of 88% in patients during VV-ECMO^15^. The pathophysiologic mechanisms underlying FXIII depletion in patients on ECMO remain incompletely understood and are likely multifactorial. Potential contributors include FXIII consumption due to systemic coagulation activation, proteolytic degradation through shear stress within the extracorporeal circuit, inflammation, and sepsis^24–26^. The fact that more than one third of study patients exhibited decreased levels of FXIII activity already at initiation of ECMO further suggests a possible role of the underlying condition. In line with our results, Moerer et al*.* reported that FXIII activity was already reduced to 37% before VV-ECMO in patients with acute respiratory distress syndrome^27^.

In our study, FXIII activity levels below the reference range (< 70%) were considered to reflect acquired FXIII deficiency. In all subjects, FXIII deficiency was classified as mild (50–69%) or moderate (30–49%), while no study patients displayed minimum FXIII activity levels < 30%. Although the definition of acquired FXIII deficiency applied in our protocol is in line with previous studies^12,18,21^, deficiencies of single coagulation factors are usually only considered clinically relevant if < 10%^28^. Despite this fact, minimum FXIII activity was correlated with both minimum haemoglobin levels and the number of pRBC transfusions in our study population, suggesting a certain additional effect of FXIII deficiency regarding bleeding complications and severity during ECMO. These findings support the physiological role of FXIII in fibrin clot stabilization and prevention of premature clot lysis^14,29,30^. Additionally, these findings highlight the importance of FXIII in the context of patient blood management and could provide a rationale for targeted FXIII monitoring as part of a standardized multimodal coagulation monitoring during ECMO^31^. In our study, minimum levels of FXIII activity were only weak predictors of major bleeding events on ECMO. Nevertheless, the identified cut-off values to discriminate between patients developing a major bleeding event or not (50.1% and 55.5%, respectively) align with reports from the trauma and cardiac surgery literature. Prior studies showed an association between FXIII activity levels < 60% and increased blood loss as well as transfusion requirements^32–35^, suggesting that, in contrast to congenital FXIII deficiency, bleeding risk seems to already be increased at much higher FXIII activity levels in critically ill patients with acquired FXIII deficiency.

The clinical relevance of FXIII monitoring and substitution remains uncertain. As this was an observational study, no definitive conclusions can be drawn from our findings regarding this issue. International recommendations on the replacement of decreased FXIII levels differ substantially. Current guidelines of the Extracorporeal Life Support Organization recommend consideration of coagulation factor correction during bleeding, but do not specifically mention FXIII thresholds^20^. Other guidelines, such as the updated European trauma guideline, suggest monitoring and replacement of FXIII in case of decreased FXIII activity levels, although precise timing and cut-off values have not been defined^36^. Similarly, the European Society of Anaesthesiology recommends monitoring FXIII in cases of ongoing or diffuse postoperative bleeding as part of a goal-directed coagulation therapy algorithm and suggests correcting FXIII levels using FXIII concentrates if they are decreased^37^. In contrast, the International Society of Thrombosis and Hemostasis recommends against the use of FXIII concentrates for managing perioperative bleeding, citing a lack of proven benefit on bleeding and transfusion requirements in acquired FXIII deficiency^38^.

To our knowledge, this is the first prospective study to systematically investigate the relationship between FXIII activity, transfusion requirements, and bleeding outcomes in adult ECMO patients. Nonetheless, several limitations need to be acknowledged. First, the observational design precludes conclusions on causal inference. Second, centre-specific practices regarding anticoagulation and haemostatic management during ECMO may limit the external validity and generalisability of our results. Additionally, the treating clinicians in our study were not blinded to FXIII results, and substitution with FXIII concentrate was permitted. This has likely influenced the course of FXIII levels in our study population and moderated the relationships between minimum FXIII activity and secondary study endpoints. Another important aspect is the potential risk of circularity between outcomes and definitions in our study. Given the fact that the ELSO definition for major bleeding events incorporates a transfusion criterion, transfusion-based based analyses may be circular.

Further, more than 40% of the study cohort were postcardiotomy ECMO patients. In most postcardiotomy patients, ECMO was started intraoperatively or the same day, reflecting the need for early postoperative support. Minimum FXIII activity did not correlate with cardiopulmonary bypass duration, and no study patient received intraoperative FXIII supplementation, thereby reducing the risk of reverse causation. Nevertheless, the potential influence of cardiopulmonary bypass priming on coagulation in terms of dilution of coagulation factors must be acknowledged. Priming types and volumes were not systematically recorded in our study, and we therefore cannot exclude residual dilutional effects of priming solution on early post-operative coagulation factor levels or occurrence of bleeding events.

In conclusion, FXIII deficiency is highly frequent in adult ECMO patients. The minimum FXIII activity was correlated with both haemoglobin levels and pRBC transfusion requirements, but not with the occurrence of major bleeding events. Larger studies are warranted to confirm optimal threshold values and determine the clinical utility of routine FXIII monitoring and targeted replacement therapy during ECMO.

## Supplementary Information

Below is the link to the electronic supplementary material.

Supplementary Material 1

Supplementary Material 2

## Data Availability

The datasets generated and analysed during the current study are not publicly available, owing to the clinical nature of the dataset and restrictions by the Ethics Committee, but are available from the corresponding author on reasonable request.

## References

[1] Combes, A. et al. Extracorporeal membrane oxygenation for severe acute respiratory distress syndrome. *N. Engl. J. Med.* **378**, 1965–1975 (2018). 29791822 10.1056/NEJMoa1800385

[2] Combes, A., Price, S., Slutsky, A. S. & Brodie, D. Temporary circulatory support for cardiogenic shock. *Lancet* **396**, 199–212 (2020). 32682486 10.1016/S0140-6736(20)31047-3

[3] Mazzeffi, M. et al. Bleeding, transfusion, and mortality on extracorporeal life support: ECLS working group on thrombosis and hemostasis. *Ann. Thorac. Surg.* **101**, 682–689 (2016). 26443879 10.1016/j.athoracsur.2015.07.046

[4] Nunez, J. I. et al. Bleeding and thrombotic events in adults supported with venovenous extracorporeal membrane oxygenation: an ELSO registry analysis. *Intensive Care. Med.* **48**, 213–224 (2022). 34921625 10.1007/s00134-021-06593-x PMC9178906

[5] Frantzeskaki, F. et al. Extracorporeal membrane oxygenation (ECMO)-associated coagulopathy in adults. *Diagnostics* **13**, 3496 (2023). 38066736 10.3390/diagnostics13233496 PMC10706340

[6] Jerrold, H. et al. ECMO-induced coagulopathy: strategic initiatives for research and clinical practice (a workshop report of the NHLBI). *Blood Vessels, Thrombosis & Hemostasis* **2**, 100064 (2025). 10.1016/j.bvth.2025.100064 PMC12320426 40766281

[7] Abrams, D. et al. Thrombocytopenia and extracorporeal membrane oxygenation in adults with acute respiratory failure: A cohort study. *Intensive Care. Med.* **42**, 844–852 (2016). 27007099 10.1007/s00134-016-4312-9 PMC5407307

[8] Granja, T. et al. Multi-modal characterization of the coagulopathy associated with extracorporeal membrane oxygenation. *Crit. Care Med.* **48**, e400–e408 (2020). 32118700 10.1097/CCM.0000000000004286

[9] Kalbhenn, J., Schlagenhauf, A., Rosenfelder, S., Schmutz, A. & Zieger, B. Acquired von Willebrand syndrome and impaired platelet function during venovenous extracorporeal membrane oxygenation: Rapid onset and fast recovery. *J. Heart Lung Transpl.* **372**, 985–991 (2018). 10.1016/j.healun.2018.03.013 29650295

[10] Hethershaw, E. L. et al. The effect of blood coagulation factor XIII on fibrin clot structure and fibrinolysis. *J. Thromb. Haemost.* **12**, 197–205 (2014). 24261582 10.1111/jth.12455

[11] Mahečić, T. T., Konosić, S., Noitz, M. & Bobinac, M. Coagulation factor XIII - last to think about?. *Blood Transfus.* **23**, 70–74 (2025). 39804745 10.2450/BloodTransfus.902 PMC11841952

[12] Traninger, A. et al. Acquired low factor XIII activity is associated with an increased need for blood transfusions in patients with gastrointestinal bleedings. *Dig. Dis. Sci.* **69**, 3894–3900 (2024). 39299997 10.1007/s10620-024-08651-y PMC11489272

[13] Kleber, C. et al. The impact of acquired coagulation factor XIII deficiency in traumatic bleeding and wound healing. *Crit. Care* **26**, 69 (2022). 35331308 10.1186/s13054-022-03940-2 PMC8943792

[14] Adam, E. H. et al. Factor XIII activity in patients requiring surgical re-exploration for bleeding after elective cardiac surgery - a prospective case control study. *J. Crit. Care* **56**, 18–25 (2020). 31805464 10.1016/j.jcrc.2019.11.012

[15] Kalbhenn, J., Wittau, N., Schmutz, A., Zieger, B. & Schmidt, R. Identification of acquired coagulation disorders and effects of target-controlled coagulation factor substitution on the incidence and severity of spontaneous intracranial bleeding during veno-venous ECMO therapy. *Perfusion* **30**, 675–682 (2015). 25823366 10.1177/0267659115579714

[16] Ito, A. et al. Acquired factor XIII deficiency in two patients with bleeding events during veno-venous extracorporeal membrane oxygenation treatment. *J. Artif. Organs* **23**, 283–287 (2020). 31834529 10.1007/s10047-019-01148-w PMC7458886

[17] Sanchez, D. et al. A pilot study to examine the effect of extracorporeal membrane oxygenation (ECMO) on plasma factor XIII levels. *Blood* **122**, 4774 (2013).

[18] Noitz, M. et al. Acquired factor XIII deficiency is common during ECMO therapy and associated with major bleeding events and transfusion requirements. *J. Clin. Med.* **12**, 4115 (2023). 37373805 10.3390/jcm12124115 PMC10299514

[19] Von Elm, E. et al. STROBE initiative the strengthening the reporting of observational studies in epidemiology (STROBE) statement: guidelines for reporting observational studies. *J. Clin. Epidemiol.* **61**, 344–349 (2008). 18313558 10.1016/j.jclinepi.2007.11.008

[20] McMichael, A. B. V. et al. 2021 ELSO adult and pediatric anticoagulation guidelines. *ASAIO J.* **68**, 303–310 (2022). 35080509 10.1097/MAT.0000000000001652

[21] Duranteau, O., Tatar, G., Demulder, A. & Tuna, T. Acquired factor XIII deficiency: A scoping review. *Eur. J. Anaesthesiol. Intensive Care.* **2**, e0035 (2023). 39916809 10.1097/EA9.0000000000000035 PMC11783664

[22] de Jager, T., Pericleous, L., Kokot-Kierepa, M., Naderi, M. & Karimi, M. The burden and management of FXIII deficiency. *Haemophilia* **20**, 733–740 (2014). 25039531 10.1111/hae.12474

[23] Laurance Lequier, G.A. et al. ELSO Anticoagulation Guideline. Extracorporeal Life Support Organization, editor. Extracorporeal Life Support Organization (ELSO); Ann Arbor, MI, USA https://www.elso.org/portals/0/files/elsoanticoagulationguideline8-2014-table-contents.pdf. (2014).

[24] Alshehri, F. S. M., Whyte, C. S. & Mutch, N. J. Factor XIII-A: an indispensable “Factor” in haemostasis and wound healing. *Int. J. Mol. Sci.* **22**, 3055 (2021). 33802692 10.3390/ijms22063055 PMC8002558

[25] Bagoly, Z., Katona, E. & Muszbek, L. Factor XIII and inflammatory cells. *Thromb. Res.* **129**, S77–S81 (2012). 22425216 10.1016/j.thromres.2012.02.040

[26] Tahlan, A. & Ahluwalia, J. Factor XIII: congenital deficiency factor XIII, acquired deficiency, factor XIII A-subunit, and factor XIII B-subunit. *Arch. Pathol. Lab. Med.* **138**, 278–281 (2014). 24476525 10.5858/arpa.2012-0639-RS

[27] Moerer, O., Huber-Petersen, J. F., Schaeper, J., Binder, C. & Wand, S. Factor XIII activity might already be impaired before veno-venous ECMO in ARDS patients: a prospective, observational single-center cohort study. *J. Clin. Med.* **10**, 1203 (2021). 33799338 10.3390/jcm10061203 PMC7999955

[28] Peyvandi, F. et al. Coagulation factor activity and clinical bleeding severity in rare bleeding disorders: results from the European Network of Rare Bleeding Disorders. *J. Thromb. Haemost.* **10**, 615–621 (2012). 22321862 10.1111/j.1538-7836.2012.04653.x

[29] Dickneite, G. et al. Coagulation factor XIII: a multifunctional transglutaminase with clinical potential in a range of conditions. *Thromb. Haemost.* **113**, 686–697 (2015). 25652913 10.1160/TH14-07-0625

[30] Guilabert, P., Asmis, L., Cortina, V., Barret, J. P. & Colomina, M. J. Factor XIII and surgical bleeding. *Minerva Anestesiol.* **88**, 156–165 (2022). 35072429 10.23736/S0375-9393.22.15772-X

[31] Žunić, M., Vreča, N. & Bevc, S. The role of factor XIII in patient blood management. *Blood Coagul. Fibrinolysis* **35**, 325–333 (2024). 39397731 10.1097/MBC.0000000000001326 PMC11462988

[32] Kaserer, A. et al. Comparison of two different coagulation algorithms on the use of allogenic blood products and coagulation factors in severely injured trauma patients: a retrospective, multicentre, observational study. *Scand. J. Trauma Resusc. Emerg. Med.* **26**, 4 (2018). 29310686 10.1186/s13049-017-0463-0 PMC5759800

[33] Innerhofer, P. et al. Reversal of trauma-induced coagulopathy using first-line coagulation factor concentrates or fresh frozen plasma (RETIC): a single-centre, parallel-group, open-label, randomised trial. *Lancet Haematol.* **4**, e258–e271 (2017). 28457980 10.1016/S2352-3026(17)30077-7

[34] Katzensteiner, M. et al. Factor XIII measurement and substitution in trauma patients after admission to an intensive care unit. *J. Clin. Med.* **11**, 4174 (2022). 35887938 10.3390/jcm11144174 PMC9317588

[35] Gödje, O., Haushofer, M., Lamm, P. & Reichart, B. The effect of factor XIII on bleeding in coronary surgery. *Thorac. Cardiovasc. Surg.* **46**, 263–267 (1998). 9885116 10.1055/s-2007-1010236

[36] Rossaint, R. et al. The European guideline on management of major bleeding and coagulopathy following trauma: sixth edition. *Crit. Care* **27**, 80 (2023). 10.1186/s13054-023-04327-7 PMC9977110 36859355

[37] Kietaibl, S. et al. Management of severe peri-operative bleeding: Guidelines from the European Society of Anaesthesiology and Intensive Care: Second update 2022. *Eur. J. Anaesthesiol.* **40**, 226–304 (2023). 36855941 10.1097/EJA.0000000000001803

[38] Godier, A., Greinacher, A., Faraoni, D., Levy, J. H. & Samama, C. M. Use of factor concentrates for the management of perioperative bleeding: guidance from the SSC of the ISTH. *J. Thromb. Haemost.* **16**, 170–174 (2018). 29168325 10.1111/jth.13893

