# Supplementary material for: Acquired factor XIII deficiency in adult patients during ECMO: a prospective observational study
Source: Sci Rep. 2025 Nov 7;15:39110. doi: 10.1038/s41598-025-26452-9 (PMC12594763; doi:10.1038/s41598-025-26452-9)
Supplement: Supplementary file 1 — Supplementary Material 1 [file 41598_2025_26452_MOESM1_ESM.docx]

**Supplementary Table S1**. Selection flow of screened patients and excluded cases

| **Exclusion criterion** | **n** | **% of all screened patients (N=146)** | **% of excluded patients (n=102)** |
| --- | --- | --- | --- |
| Age <18 years | 44 | 30.1% | 43.1% |
| Pre-existing hereditary haemorrhagic/thrombophilic disorder | 4 | 2.7% | 3.9% |
| CARL-ECPR | 6 | 4.1% | 5.9% |
| ECMO duration <24 hours | 10 | 6.8% | 9.8% |
| ECMO as temporary RVAD (after LVAD) | 6 | 4.1% | 5.9% |
| Consent refused | 12 | 8.2% | 11.8% |
| Other reasons | 20 | 13.7% | 19.6% |
| **Total excluded** | **102** | **69.9%** | — |
| **Included in study/analysis** | **44** | **30.1%** | — |

CARL, controlled automated reperfusion of the whole body; ECMO, extracorporeal membrane oxygenation; ECPR, extracorporeal cardiopulmonary resuscitation; RVAD, right ventricular assist device.

**Supplementary Table S2.** Distribution of bleeding locations and major bleeding criteria

| **ELSO major bleeding criterion** | **n** | **% of patients with major bleeding events during ECMO** |
| --- | --- | --- |
| Transfusion criterion (≥10 mL/kg in 24 hours) | 24 | 72.7% |
| Hb drop ≥2 g/dL within 24 hours | 17 | 51.5% |
| Cannulation site bleeding | 16 | 48.5% |
| Surgical revision | 8 | 24.2% |
| Pulmonary bleeding | 8 | 24.2% |
| Pericardial bleeding | 6 | 18.2% |
| ENT bleeding | 5 | 15.2% |
| Hemothorax | 4 | 12.1% |
| Intracranial hemorrhage | 2 | 6.1% |
| Gastrointestinal bleeding | 2 | 6.1% |
| Urethral bleeding | 1 | 3.0% |
| Liver bleeding | 1 | 3.0% |
| Retroperitoneal bleeding | 0 | 0.0% |
|  |  |  |
|  |  |  |

ECMO; extracorporeal membrane oxygenation, ELSO, Extracorporeal Life Support Organization; ENT, ear-nose-throat; Hb, haemoglobin.

**Supplementary Table S3.** Day of ECMO therapy for first major bleeding event (overall and by site)

| **Bleeding Site** | **n** | **%** | **Day of first major bleed, median (IQR)** |
| --- | --- | --- | --- |
| Overall (first major bleeding event) | 33 | 100 | 1.0 (0.5–3.0) |
| Cannulation site | 16 | 48.5 | 1.0 (0.0–2.2) |
| Pulmonary bleeding | 8 | 24.2 | 1.0 (1.0–1.0) |
| Pericardial bleeding | 6 | 18.2 | 1.0 (1.0–3.2) |
| ENT bleeding | 5 | 15.2 | 0.5 (0.0–1.2) |
| Hemothorax | 4 | 12.1 | 4.5 (3.0–5.0) |
| Intracranial hemorrhage | 2 | 6.1 | 3.0 (3.0–3.0) |
| Gastrointestinal bleeding | 2 | 6.1 | 6.5 (5.8–7.2) |
| Urethral bleeding | 1 | 3 | 1.0 (1.0–1.0) |
| Liver bleeding | 1 | 3 | 1.0 (1.0–1.0) |
| Retroperitoneal bleeding | 0 | 0 | - |

ENT, ear-nose-throat.
