# Supplementary material for: Acquired factor XIII deficiency in adult patients during ECMO: a prospective observational study
Source: Sci Rep. 2025 Nov 7;15:39110. doi: 10.1038/s41598-025-26452-9 (PMC12594763; doi:10.1038/s41598-025-26452-9)
Supplement: Supplementary file 2 — Supplementary Material 2 [file 41598_2025_26452_MOESM2_ESM.docx]

**FIGURE LEGENDS SUPPLEMENTARY FILES**

**
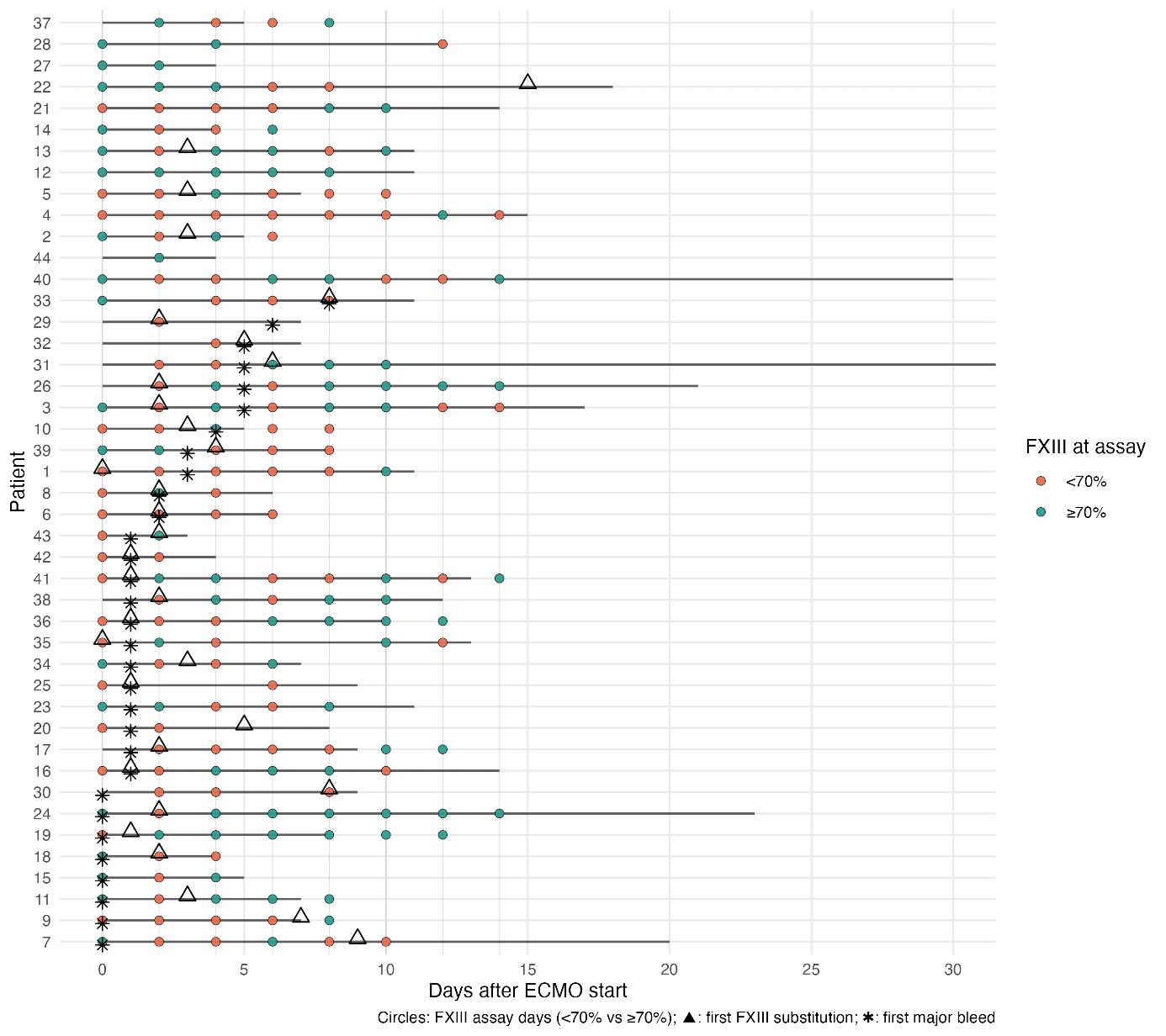
**

**Supplementary Figure S1.** Swimmer plot of per-patient follow-up from ECMO cannulation.

Horizontal bars show ECMO duration. Circles indicate FXIII assay day. The triangle (▲) marks the first FXIII substitution. Asterixis (✱) marks the first major bleeding event.
